# Observing Climate Change Impacts on European Forests: What Works and What Does Not in Ongoing Long-Term Monitoring Networks

**DOI:** 10.3389/fpls.2017.00629

**Published:** 2017-04-25

**Authors:** Filippo Bussotti, Martina Pollastrini

**Affiliations:** Laboratory of Environmental and Applied Botany, Department of Agri-Food Productions and Environmental Science, University of FlorenceFlorence, Italy

**Keywords:** crown dieback, crown defoliation, ICP forests, ecosystem functions, ecosystem services, national forest inventories, tree growth, tree mortality

## Introduction

Ecosystem services of forests are related to specific functions, such as growth, photosynthesis, regeneration, element cycling, soil formation, biodiversity holding, and so on. For this reason, a wide “bouquet” of attributes must be taken into consideration to evaluate the overall functionality of forests and their capacity to provide ecosystem services. These attributes may be considered “proxies” for specific relative ecosystem functions and services. The growth of trees and standing biomass is connected not only to the economic value of the forest (provisional services) but also to carbon sequestration and climate regulation (regulatory services). The overall canopy closure (i.e., leaf area index—LAI) can be considered a proxy for stand productivity (Gower et al., 1999) and stand transpiration (Yan et al., 2012), trees' capacity of uptake and filtration of air pollutants (Janhäll, 2015), and element cycling (Burton et al., 1991) as well for soil conservation through the regulation of heavy rain impacts (Park and Cameron, 2008). Parameters related to stand structure, species composition, and diversity may be considered indicative of the capacity of regeneration, to support game and wildlife and to host beneficial organisms against parasites. Some of these attributes are currently assessed in national forest inventories, and data can be useful to map the extension and level of related ecosystem services.

The ecosystem functions and services of forests can be affected by the harshening of environmental conditions. Climate change impacts on forests is the result of complex interactions between meteorological factors and soil conditions (Barbeta et al., 2015), pathological agents (Wermelinger et al., 2007), forest fires (Flannigan et al., 2000), and environmental pollution (De Vries et al., 2014). The action of these co-occurring factors contributes to tree mortality and the reduction of canopy cover, as well-changes in stand composition and diversity (Millar and Stephenson, 2015). These effects can be evaluated by comparing subsequent forest inventories; however, the long period of time elapsing between two successive surveys can make them scarcely applicable if our purposes are to detect and monitor in time and space the impacts of climate change on forest ecosystem structure, functionalities and services provided.

In Europe and North America are currently implemented programs for forest health assessment. The European ICP Forests program (www.icp-forests.net) consists of two networks of monitoring plots (extensive and intensive) where various monitoring activities are repeated at different temporal bases. These networks were designed to assess the effects of transboundary air pollution and atmospheric depositions (De Vries et al., 2014) and represent the most important tool to assess changes in forest ecosystems health condition at national and European scales. A change of perspective, from atmospheric pollution and deposition to multiple stress pressure related to climate change, suggests a revaluation of the overall structure of the surveys and of the indicators adopted to observe the impacts on forests. The aim of this study is to verify whether, and to what extent, the current European forest health monitoring program is suitable for assessing the impacts of new global environmental factors, and give suggestions to enhance its informative potential.

## Impacts at tree level: tree growth and crown defoliation

The overall impacts of environmental stress on trees can be summarized with their effects on growth. Tree growth is a key parameter for evaluate the ability of forests to mitigate climate change and provide ecosystem services (Bonan, 2008). Forest health, however, is commonly assessed by means of crown defoliation (Michel and Seidling, 2014). Defoliation is a visual estimate of the relative amount of foliage loss of the target tree compared with that of the reference standard tree. Crown defoliation has been assessed extensively since the 1980s, and the trends recorded are assumed to be correlated with the effects of environmental stress, such as air pollution and climate change (Van Leeuwen et al., 2000). Although no clear relationships were found between defoliation and ecological factors at European level, local studies evidenced the impacts of ozone pollution (Augustatis and Bytnerowicz, 2008); extreme drought and heat weaves (Carnicer et al., 2011) and fluctuating climatic conditions (Ferretti et al., 2014). Intuitively, defoliation may affect growth through the reduction of the photosynthetic surface, but different ecophysiological processes (e.g., better exploitation of sunlight from the foliage in the inner part of the crown, increase of photosynthetic efficiency in the remaining foliage) can compensate for the loss of leaves. Growth reduction has been verified only for the most defoliated pine species (*Pinus sylvestris* L.) in Spain (Sánchez-Salguero et al., 2012), Italy (Castagneri et al., 2015), and Lithuania (Augustatis and Bytnerowicz, 2008).

## Changes and dynamisms in forests subjected to climate change

According to Millar and Stephenson (2015), forest cover under climatic change undergoes a progressive dieback of the crown and mortality of trees. During the period of decline, a regeneration of trees of different species, or of different genotypes of the same species, will occur. The decline of canopies induced by climate change may trigger a vegetational dynamism, with a temporary success of the early-successional tree species. In a longer time span, the regeneration of a “definitive” (mature) tree cover is supposed to be ensured by more xerophytic species coming from proximal areas (e.g., from lower elevation). This new generation of trees is supposed to be better adapted to the environmental condition of the previous one. The functions and services of forests are assumed to decrease during the canopy-decline period, but they are likely to be restored when the “new forest” is well-established. The equilibrium of the forest, adapted to a new environment, implies probably a different level of ecosystem functions and services, with respect to that which is provided by forests before the action of disturbing factors. Adaptation to drier conditions determines slower growth rates, lower foliar mass, reduced regeneration rate, and so on. These processes are ongoing in some parts of Europe and can be observed, for example, in *P. sylvestris* stands in the Valais (Switzerland) and the Italian Western Alps, where *Quercus pubescens* Willd. is going to replace *P. sylvestris* in the driest conditions (Rigling et al., 2013). The changes in the forest structure and species composition are associated with several events and signals, among which the most significant, due to its consequences, is the drought-induced tree mortality (Allen et al., 2015) and insect attacks in trees weakened by drought (Wermelinger et al., 2007; Anderegg et al., 2015).

Large spatial-scale events of drought-induced tree mortality have been registered in Europe and North America (Anderegg et al., 2013). Tree death is generally preceded by a severe decline of the growth and the dieback of a large part of the crown (Sánchez-Salguero et al., 2012). Dobbertin and Brang (2001) probed that the adoption of “defoliation” improves models to predict the mortality of trees in national forest inventories. Substantial reduction of foliar mass is assumed reflect the loss of photosynthates and compromises carbon allocation and tree growth (Garcia-Forner et al., 2016). According to Grote et al. (2016), big trees are especially subjected to water shortage and more likely to die than small trees, but Ruiz-Benito et al. (2013) and Van Gunst et al. (2016) found that in a “competitive” environment, as far as water resources are concerned, small trees are more likely to die.

A different scenario suggests the persistence of the current tree species, but with changes in the forest structure (i.e., reduction of tree density) to withstand the reduction of water resources. The “local evolution,” or adaptation, of dominant tree species is promoted by the selection of more adapted individuals, that are those bearing the most suitable genotypes (Savolainen et al., 2007). The local evolution of species as a result of climate change is achieved by taking advantage of the existing genetic variability in natural populations. Increasing interest is devoted to epigenetic acclimation processes (Barbeta et al., 2013) and the so-called memory effect (Crisp et al., 2016), which may be responsible for the progressive reduction of the negative effects of severe drought on tree physiology and survival (Liu et al., 2015).

Tree diversity enhances growth and stabilizes productivity at stand level and on individual trees (Jucker et al., 2014; Liang et al., 2016), and it is supposed to modify the impacts of stress factors through “plant-to-plant” interactions. Such interactions may be either negative (competition) or positive (facilitation). According to the “stress gradient” hypothesis (He and Bertness, 2014), positive interactions are more frequent than negative ones in the poorest sites, where resources are scarce.

## What works and what does not

Changes in the health and physiological conditions of trees are assumed to provide insights for the prevision of changes in ecosystems and may represent an alarm signal to interpret the dynamics of acclimation and/or adaptation processes of forests in new environmental conditions. Moreover, responses at tree and stand levels are influenced by stand structure, tree species composition, genomic features and overall diversity. Therefore, adopting a holistic view in the interpretation of interactions between tree health and all the components and processes of the ecosystem is necessary and more realistic.

The greatest usefulness of the extensive forest monitoring network in Europe is its very existence, as well as the existence of expert groups of scientists and technicians at national and European levels and a set of consolidated methods and manuals that make the assessed parameters (i.e., defoliation) reliable and generally accepted. What it needs is the introduction of concepts and indicators suitable for evaluating the mechanisms and processes performed by trees to withstand new environmental challenges owing to climate change. This integration of the current forest monitoring approach implies a substantial improvement of the diagnostic capacity of tree health indicators, combining the traditional visual assessment with more effective morphological and physiological indicators (Bussotti and Pollastrini, 2015).

The data series of ICP Forests (Level I, Timmermann et al., 2016), available from'90 in the past century, suggest a substantial stability (or slightly increasing defoliation) of crown conditions at the European level of some of the most diffuse tree species (*P. sylvestris, Picea abies* (L.) Karst., *Fagus sylvatica* L.), alongside a progressive worsening of crown conditions of Mediterranean species. Local events of mortality (Bussotti et al., 2014, 2015), with impact at the regional level, are scarcely captured in the current forest monitoring activities. Therefore, for management purposes, locally intensifying the network to capture the most critical tree species and/or ecological conditions may be useful. This result can be achieved by involving local communities, as well as appealing to the so-called citizen science (McKinley et al., 2017).

## Conclusions: what we can do

To make the current terrestrial surveys able to capture the changes in forest ecosystems due to climate change, we need to shift the focus from the conditions of individual trees to the demography of the community. Mortality (including small trees, suckers, and understory woody vegetation) and regeneration are therefore key parameters to predict and interpret changes in tree species composition, local evolution, and possible desertification processes. Before tree death physiological stress conditions occur. These changes in plant health status, can be effectively assessed by foliar parameters, such as carbon isotope composition (that is proxy of drought stress, (Farquhar et al., 1982), and of carbon sequestration strategies of plants), chlorophyll fluorescence analysis, as indicator of overall plant stress conditions (Bussotti et al., 2010), and leaf morphology parameters (e.g., SLA, that indicates photosynthetic acclimation and vulnerability to stress, Bussotti, 2008). Tree ring analysis, moreover, is highly desirable to explore tree growth responses in relation to resilience, mortality and foliage loss (Lloret et al., 2011). A first list of the actions needed to improve the informative potential of extensive monitoring surveys in forest ecosystems is provided in Table 1.

**Table 1:** **Summary of the main features of the current extensive terrestrial survey in Europe (ICP Forests, Level I), and proposed additions to make the survey more effective to capture the effects of climate change**.

**Current surveys**	**Proposed surveys**
**GENERAL OBJECTIVES**	
Tree-based survey	Stand (forest population/community)-based survey
To assess the conditions of trees in relation to air pollution, atmospheric deposition, and other environmental stress	To assess the changes in the structure and species composition of forests under climate change
**SAMPLING DESIGN**	
Spatial distribution according to a regular grid	Regular spatial distribution can be locally intensified to capture the most critical situations. Contribute of local communities and citizens
Assessment is done every year.	Assessment may be done at multi-year basis (e.g., every 3–5 years)
**INDICATORS**	
Mortality of trees with diameter at breast height (DBH)>10 cm	Mortality of trees and woody vegetation, including suckers and understory
Crown conditions (defoliation and symptoms) of trees with DBH>10 cm	Foliar analysis (Chlorophyll content and fluorescence, carbon isotope composition, leaf morphology) combined with crown condition assessment
Measurement of DBH every 5 year	Measurement of DBH combined with tree ring analysis to assess responses and resilience to severe weather events
	Leaf Area Index evolution
	Regeneration
**RESULTS REPORTING**	
Percent of trees with defoliation over a certain threshold	Temporal and spatial changes in structure and composition of forest in relation to environmental factors
Percent of symptomatic trees	

Establishing close relationships and exchanges between different levels of intensity in monitoring programs is desirable. Intensive monitoring networks, observational comparative plots (Baeten et al., 2013; von Gadow et al., 2016) and experimental plots (ecosystem manipulation, Perry and Troelstrup, 1988) are relevant for the assessment and validation of the feasibility of the proxies to be extensively assessed and their ecological and physiological significance.

## Author contributions

FB and MP contributed equally to the discussion of the topic.

### Conflict of interest statement

The authors declare that the research was conducted in the absence of any commercial or financial relationships that could be construed as a potential conflict of interest.
